# A Quality Improvement Bundle to Reduce Ambulatory CLABSI: The Importance of a Multidisciplinary Team

**DOI:** 10.1097/pq9.0000000000000500

**Published:** 2021-09-02

**Authors:** Megan E. Gabel, Samantha W. Neumeister, Jeffrey M. Meyers, Jan A. Schriefer

**Affiliations:** From the Department of Pediatrics, Golisano Children’s Hospital at University of Rochester Medical Center, Rochester, N.Y.

## Abstract

Children’s Hospitals’ Solutions for Patient Safety (SPS) is a network of over 140 children’s hospitals who share the vision of working together to eliminate serious harm across all pediatric hospitals. The SPS network is built on the fundamental belief that by sharing successes and failures transparently and learning from one another, children’s hospitals can achieve their goals more effectively and quickly than working alone. Each year, SPS hosts National Learning Sessions to which members are invited to submit abstracts describing relevant safety research or improvement work. The following abstracts were among the top submitted for the SPS Spring 2021 National Learning Session.

## Background:

Intestinal failure patients with central line-associated bloodstream infections (CLABSI) have increased morbidity and mortality, reported at 12%–25%. This often-preventable complication accounts for at least 50% of the hospitalizations for this patient population.

## Objectives:

Our initial rate of home-acquired CLABSI in home parenteral nutrition dependent patients was 7.6/1000 catheter days. Our aim was to decrease the central line infection rate in this population by 25% through the development and implementation of an enhanced care bundle.

## Methods:

We established a multidisciplinary team, including a quality improvement coach, a nurse, and a physician. The design of our central venous catheter maintenance bundle was based on interview of families and literature review. With the guidance of fishbone and key driver diagrams, we created a central venous catheter care tote, including additional supplies and improved educational materials.

## Results:

**Fig. 1.**
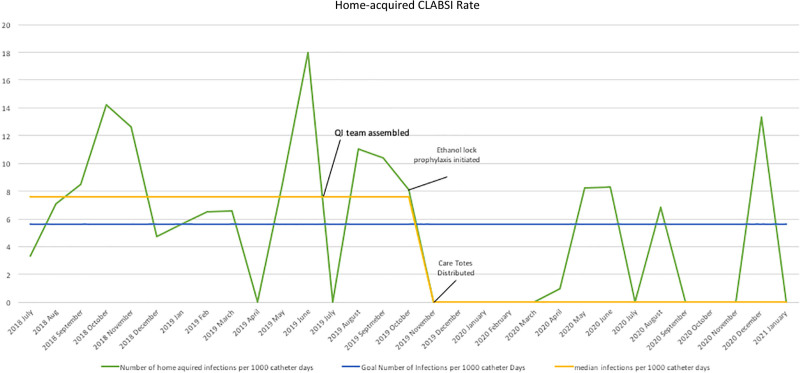
Run chart of home acquired CLABSI rate in our parenteral-nutrition-dependent population.

The central venous catheter bundle was implemented in October 2019, following which a special cause variation was noted. Our median monthly infection rate decreased from 7.6 to 0 infections per 1000 catheter days; the average rate declined from 8.0 to 2.5. As a result, eight fewer home-acquired CLABSIs are occurring each year in our patient population. Our data is summarized in Figure 1.

## Conclusions/Implications:

This study highlights the importance of quality improvement in the prevention of home-acquired CLABSI, and underscores the value of multidisciplinary teams in the improvement process.

